# EDTA and NTA Effectively Tune the Mineralization of Calcium Phosphate from Bulk Aqueous Solution

**DOI:** 10.3390/biomimetics2040024

**Published:** 2017-12-13

**Authors:** Doreen Hentrich, Klaus Tauer, Montserrat Espanol, Maria-Pau Ginebra, Andreas Taubert

**Affiliations:** 1 Institute of Chemistry, University of Potsdam, 14476 Potsdam, Germany; hentrich@uni-potsdam.de; 2 Max Planck Institute of Colloids and Interfaces, 14476 Potsdam, Germany; klaus.tauer@mpikg.mpg.de; 3 Biomaterials, Biomechanics and Tissue Engineering Group, Department of Materials Science and Metallurgy, Universitat Politècnica de Catalunya, Avinguda d’ Eduard Maristany 10-14, 08019 Barcelona, Spain; Montserrat.Espanol@upc.edu (M.E.); maria.pau.ginebra@upc.edu (M.-P.G.); 4 Barcelona Research Centre in Multiscale Science and Engineering, Universitat Politècnica de Catalunya, 08019 Barcelona, Spain

**Keywords:** biomineralization, biomimetic mineralization, calcium phosphate, NTA, EDTA, precipitation, brushite, hydroxyapatite

## Abstract

This study describes the effects of nitrilotriacetic acid (NTA) and ethylenediaminotetraacetic acid (EDTA) on the mineralization of calcium phosphate from bulk aqueous solution. Mineralization was performed between pH 6 and 9 and with NTA or EDTA concentrations of 0, 5, 10, and 15 mM. X-ray diffraction and infrared spectroscopy show that at low pH, mainly brushite precipitates and at higher pH, mostly hydroxyapatite forms. Both additives alter the morphology of the precipitates. Without additive, brushite precipitates as large plates. With NTA, the morphology changes to an unusual rod-like shape. With EDTA, the edges of the particles are rounded and disk-like particles form. Conductivity and pH measurements suggest that the final products form through several intermediate steps.

## 1. Introduction

The increasing age of the world population leads to an increasing demand for materials for hard tissue repair. Many of these materials are based on calcium phosphate (CP) [1,2,3,4,5] and, for a biomaterial to work properly, it is necessary to tune and tailor synthetic CP-based biomaterials for a specific (medical) application. To that end, it is necessary to understand and control CP mineralization during synthesis. Ideally the resulting synthetic materials are similar to natural hard tissue [6,7] or precursors to biological minerals [8,9]. The crystal phase, crystal morphology, and crystal size of synthetic CP precipitates can be influenced by additives [7,8,9,10,11] such as polymers [12,13,14,15,16,17,18,19,20], surfactants [21], amino acids [22], poly(amino) acids [20,23,24,25,26,27,28,29,30,31,32,33], or poly(cations) [32,34,35,36,37,38,39,40,41].

Using poly(acrylic acid) (PAA) with different molar masses (2000 and 250,000 g/mol), Bigi et al. [14] demonstrated that the molecular weight of the additives plays a decisive role in CP precipitation from aqueous solution: PAA adsorbs on octacalcium phosphate (OCP) surfaces and this adsorption prevents the splitting of the OCP crystals, which in turn suppresses the transformation of OCP to hydroxyapatite (HAP). According to the authors of this study, the lower molecular weight PAA adsorbs more efficiently to the OCP (100) crystal surface. This is likely due to a better packing of the shorter chains on the OCP crystal surface. As a result, PAA with a molecular weight of 2000 g/mol inhibits the OCP/HAP transformation more effectively [14]. This study shows that low(er) molecular weight compounds have a pronounced effect on CP mineralization from aqueous solution. In spite of this, many recent studies focus on higher molecular weight additives [8,9].

Some studies, however, show that small molecules like citrate affect CP mineralization [42,43,44,45,46,47]. CP mineralization in the presence of citrate proceeds via an amorphous CP (ACP) precursor phase, which then directly transforms to HAP; this transition is accompanied by a pH drop from 8.5 to 6.7 during the first 5 min [44]. In the absence of citrate, dicalcium phosphate dihydrate (DCPD, brushite) first transforms to OCP and finally to HAP via processes following the Ostwald step rule; this series of transitions is accompanied by a significant overall pH drop from 8.5 to 4.9 over ca. 25 min [7,48,49,50]. The reason for the difference between the mineralization with and without citrate is the stabilization of the ACP precursor by citrate adsorption on the surface of the ACP particles. Similar to another study [42,44], the authors ruled out that a decreasing number of free calcium ions by complexation trough the citrate is the reason for the different growth process. In fact, this study specifically claims that low molecular weight molecules could be valuable and effective growth modifiers in terms of size, shape, and crystal phase selection for CP precipitation.

Chelating agents like ethylenediaminotetraacetic acid (EDTA) also affect CP formation [51,52,53,54,55,56]. These studies mostly exploit the decomposition of Ca–EDTA complexes and CP synthesis was performed under hydrothermal conditions [51,52,53]. Arce et al. [53] synthesized HAP by decomposition of Ca–EDTA complexes in the presence of phosphate at 100, 120, and 140 °C. At pH 5, the authors obtained plate-like monetite (dicalcium phosphate anhydrous, DCPA) crystals at lower temperatures. At higher temperatures, the crystals transform to a mixture of DCPA and HAP. At pH 7 and 9 elongated HAP crystals form. At pH 7 the crystals have a size of 5–10 µm independent of the temperature and at pH 9 the sizes change with increasing temperature to form larger crystals [53]. Similarly, Kalita et al. [56] made nanocrystalline (12 nm) HAP particles with different morphologies using microwave irradiation.

The studies just described use rather harsh reaction conditions, a situation which is not always desired. As a result, the current study evaluates the effects of two low molecular weight additives, nitrilotriacetic acid (NTA) and EDTA, on CP mineralization from bulk aqueous solution under mild conditions. Both additives form chelate complexes and contain different numbers of carboxyl groups, which can interact with the CP precipitate; a strong interaction with calcium ions in solution and the surface of the CP precipitates (and hence a tight control over the mineral formation) can therefore be expected. Although the reaction medium per se, an aqueous solution, is not necessarily a true biomimetic environment, the effects observed in the study clearly have a biomimetic aspect. They provide information on how CP precipitation can very easily be tuned; they should thus provide a new pathway into the development of advanced bio- and biomimetic materials using a very mild process.

## 2. Materials and Methods

### 2.1. Chemicals

Calcium nitrate tetrahydrate (≥99%, p.a., ACS; Carl Roth GmbH, Karlsruhe, Germany), diammonium hydrogen phosphate (98%; Alfa Aesar, Karlsruhe, Germany), ethylenediaminotetraacetic acid disodium salt dihydrate (≥99%, USP; Carl Roth GmbH), MilliQ^®^ water (0.055 µS/cm; Merck-Millipore, Darmstadt, Germany), nitrilotriacetic acid trisodium salt monohydrate (≥98%; Sigma-Aldrich, Darmstadt, Germany), hydrochloric acid (37%; Merck, Darmstadt, Germany), sodium hydroxide (99%, p.a., ISO; Carl Roth GmbH) were used as received. EDTA and NTA purity was ascertained with nuclear magnetic resonance (NMR) and infrared (IR) spectroscopy and elemental analysis.

#### 2.1.1. Nitrilotriacetic Acid Trisodium Salt Monohydrate

^1^H-NMR (300 MHz, D_2_O): δ (ppm) = 3.11 (s, 6H). ^13^C-NMR (300 MHz, D_2_O): δ (ppm) = 181.1, 59.11. IR (ATR) ν = 505, 521, 532, 582, 633, 648, 729, 754, 806, 843, 920, 947, 955, 962, 984, 995, 1003, 1062, 1099, 1132, 1140, 1242, 1257, 1284, 1327, 1340, 1361, 1404, 1450, 1473, 1506, 1572, 1674, 2873, 2906, 3163, 3330 cm^−1^. Elemental analysis (C_6_H_8_NO_7_Na_3_): calculated (found) C 26.20% (26.25%); H 2.93% (2.85%); N 5.09% (5.09%).

#### 2.1.2. Ethylenediaminotetraacetic Acid Disodium Salt Dihydrate

^1^H-NMR (300 MHz, D_2_O): δ (ppm) = 3.88 (s, 8H); 3.66 (s, 4 h). ^13^C-NMR (300 MHz, D_2_O): δ (ppm) = 170.7, 58.0, 51.6. IR (ATR) ν = 444, 494, 550, 625, 704, 816, 899, 920, 939, 957, 1020, 1053, 1134, 1196, 1225, 1267, 1292, 1315, 1356, 1392, 1475, 1620, 1672, 2729, 2779, 3028, 3381, 3525 cm^−1^. Elemental analysis (C_10_H_18_N_2_Na_2_O_10_): calculated (found) C 32.27% (32.28%); H 4.87% (5.01%); N 7.53% (7.55%).

### 2.2. Mineralization

For all mineralization experiments, a calcium and phosphate concentration of 20 mM was used. NTA and EDTA (concentrations: 5, 10, and 15 mM) were used as mineralization additives. First, 50 mL of a 1 M Ca(NO_3_)_2_ (11.808 g of Ca(NO_3_)_2_·4H_2_O in 50 mL of MilliQ^®^ water) and (NH_4_)_2_HPO_4_ (6.602 g (NH_4_)_2_HPO_4_ in 50 mL of MilliQ^®^ water) stock solutions were produced. The final 40 mM solutions were then prepared via dilution of these stock solutions with appropriate amounts of deionized water. The pH was adjusted by addition of aqueous HCl or NaOH during the preparation of these solutions. The additive (NTA or EDTA) was directly dissolved in the Ca(NO_3_)_2_ solutions followed by pH adjustment. To start the mineralization, 50 mL of the 40 mM (NH_4_)_2_HPO_4_ solution were rapidly added to 50 mL of the 40 mM Ca(NO_3_)_2_ solution (with or without the additive), yielding an overall 20 mM solution with respect to both calcium and phosphate (P_i_). Mineralization time was 4 h under constant stirring at 800 rpm (Thermo Scientific Cimarec i advanced multipoint 6, Langenselbold, Germany). All precipitates were centrifuged, washed three times with 40 mL of MilliQ^®^ water, and dried in an oven at 37 °C. All reactions were performed in 100 mL polypropylene vials with screw cap. Reaction temperature was 20 ± 1 °C. Table 1 summarizes the mineralization reaction parameters.

### 2.3. Attenuated Total Reflectance–Fourier Transform Infrared Spectroscopy

Attenuated total reflectance–Fourier transform infrared (ATR–FTIR) spectra were recorded on a Nicolet 6700 Thermo Scientific spectrometer (Waltham, MA, USA) using a Thermo Scientific smart orbit (diamond crystal) and an MCT/A detector from 400 to 4000 cm^−1^. Measurements were done using Omnic version 6.2 software (Thermo Electron Corp., Waltham, MA, USA) and each spectrum was recorded with 32 scans with a measuring time of 32 s and a resolution of 4 cm^−1^. Prior to the measurements, the samples were ground and directly deposited on the diamond crystal.

### 2.4. Elemental Analysis

Elemental analysis was done on a Vario EL III (Elementar, Langenselbold, Germany).

### 2.5. Nuclear Magnetic Resonance Spectroscopy

^1^H- and ^13^C-NMR spectra were recorded on a Bruker Avance 300 spectrometer (Billerica, MA, USA). Chemical shifts (δ) are in ppm using residual signals from the deuterated solvents for reference.

### 2.6. Scanning Electron Microscopy

Scanning electron microscopy (SEM) measurements were done on a JEOL JSM-6510 microscope (Freising, Germany). The dry, as-synthesized precipitates were deposited on Al SEM stubs with carbon glue pads and sputter-coated with Pd/Au. Images were acquired at 1 or 2 kV to avoid sample charging.

### 2.7. X-ray Powder Diffraction

X-ray diffraction (XRD) measurements were performed on a PANalytical Empyrean diffractometer (EA Almelo, The Netherlands) between 4 and 70° 2θ with a step size of 0.0131° (secondary monochromator, λ = 1.5408 Å (CuK_α_), automatic divergence aperture, and rotating sample holders). The samples were ground and deposited on silicon single crystal disc sample holders.

### 2.8. Conductivity Measurements

Conductivity measurements were done with a PP1042 probe and a CDM 83 instrument (Radiometer, Copenhagen, Denmark).

### 2.9. pH Measurements

pH measurements were done with a MultiLab 540 pH meter (WTW, Weilheim, Germany) and a SenTix 41 pH electrode (WTW, Weilheim, Germany) in 50 mL polypropylene beakers with screw cap. Mineralization was done as described above, but the total liquid volume was 30 instead of 100 mL. Before mineralization was started, the pH and the conductivity of the 40 mM Ca(NO_3_)_2_ solution (including the desired additive concentration) was measured for 5 min. At pH 6 the pH and conductivity were measured over 140 min for NTA and over 60 min for EDTA, at pH 7 and 8 over 30 min, and at pH 9 over 170 min for NTA and 70 min for EDTA. Table 2 summarizes the concentrations, masses, and volumes for the pH and conductivity measurements.

## 3. Results

### 3.1. Calcium Phosphate Mineralization with NTA

**Scheme 1:**
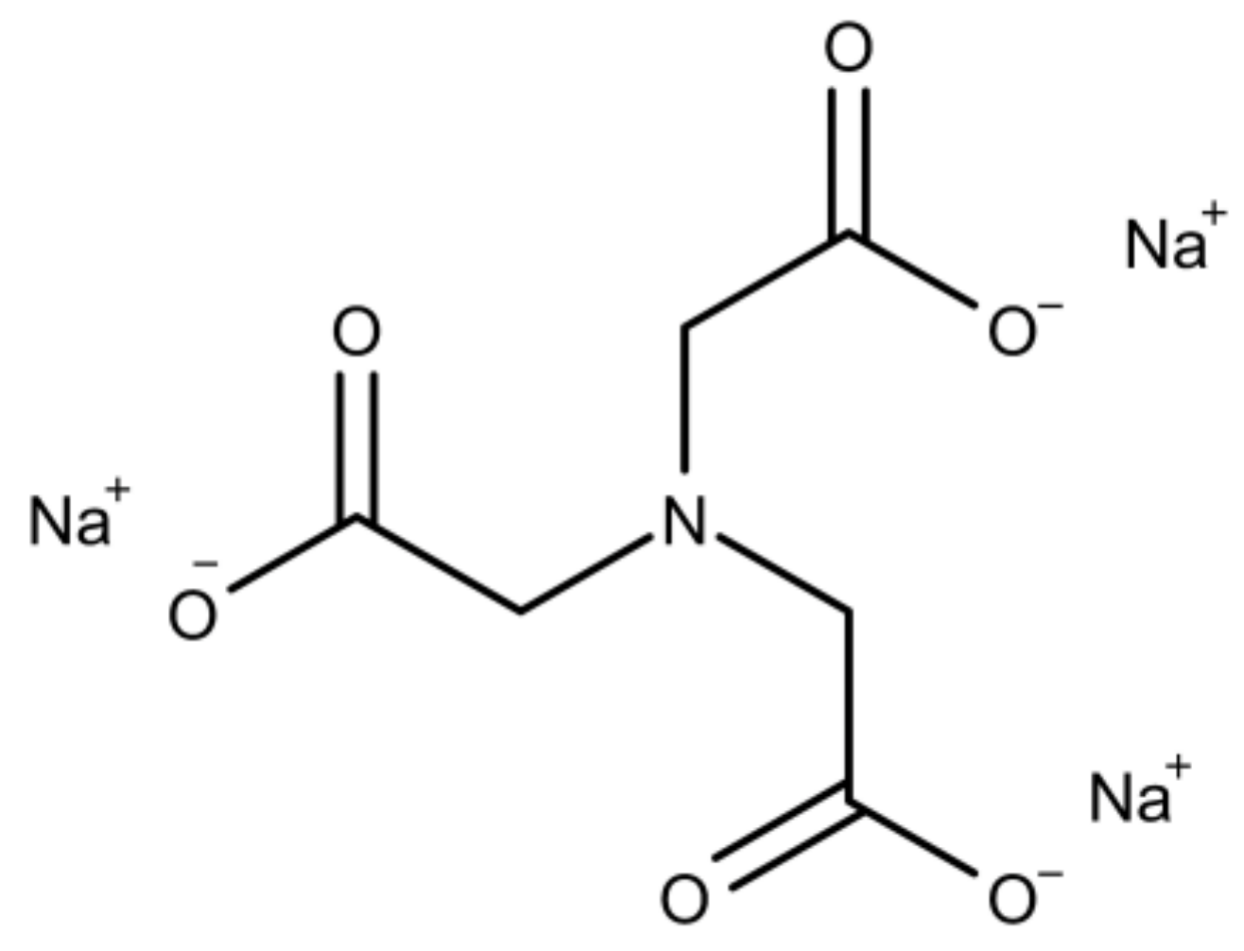
Chemical structure of NTA [59].

NTA is a tripodal tetradentate trianionic ligand and chelating agent (Scheme 1) and forms a stable complex with calcium [57,58]. Calcium phosphate mineralization was performed in bulk solution between pH 5 and 9 in the presence (5, 10, and 15 mM) and in the absence (0 mM) of NTA over 4 h. The samples mineralized in the absence of NTA are the control samples.

**Figure 1:**
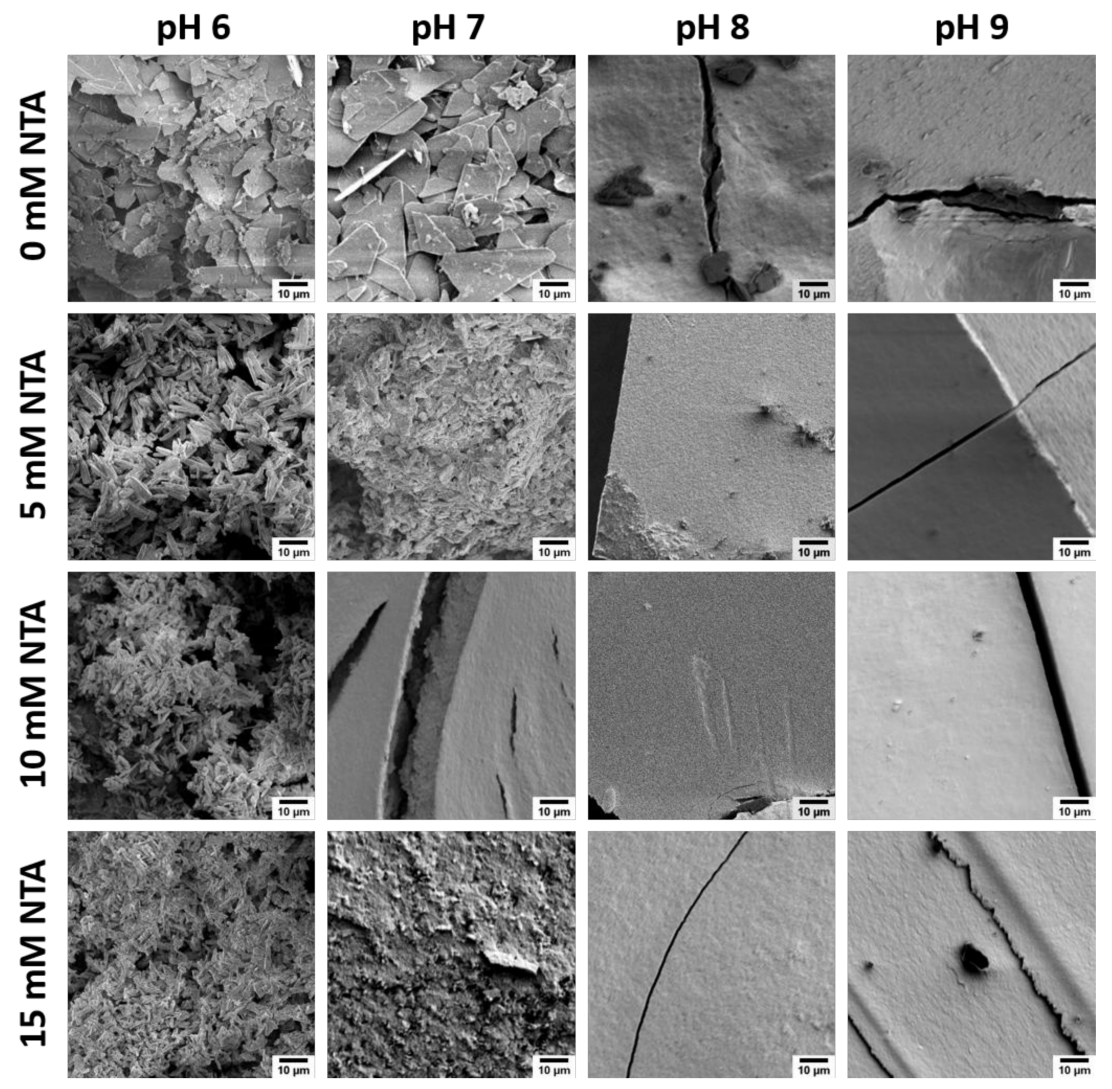
Representative scanning electron microscopy (SEM) images of CP precipitated in the absence (control samples) and presence of NTA at different pH. Higher magnification images of the aggregated blocks are shown in the Appendix A, Figure A1.

Figure 1 shows SEM images of the precipitates. The control samples grown without additive at pH 6 consist of plates of different sizes. The smaller plates are between 5 and 7 µm and are probably in part fragments of larger plates with sizes of 13–16 µm. The samples grown at pH 6 with NTA mainly contain rods. The rod length decreases with increasing NTA concentration, from 5 to 11 µm at 5 mM, to 3–7 µm at 10 mM, and to 2–6 µm at 15 mM of NTA.

The control samples obtained at pH 7 are similar to the control samples at pH 6 and mostly show plates. These plates are much bigger than those observed at pH 6 and have sizes between 17 and 23 µm. Smaller plates are also visible, but their concentration is much lower when compared to pH 6. Similar to pH 6, rods are the predominant particle morphology also at pH 7 at low NTA concentrations of 5 mM. These rods are less well formed and closely aggregated; this makes a size estimation difficult. With higher NTA concentrations of 10 mM, neither plates nor rods are visible. Instead, the morphology resembles micrometer-sized dense blocks, which appear to break during the drying process. Higher magnifications show that the blocks are made of small nanoparticles with sizes between 10 and 100 nm. The samples grown at pH 7 with 15 mM of NTA also consist of dense blocks, but these blocks have a rougher surface compared to the products grown at 10 mM of NTA.

Precipitation at pH 8 or 9 leads to control samples containing dense blocks and small plates on the surface of the blocks. All samples grown in the presence of NTA again show dense blocks independent of the NTA concentration.

**Figure 2:**
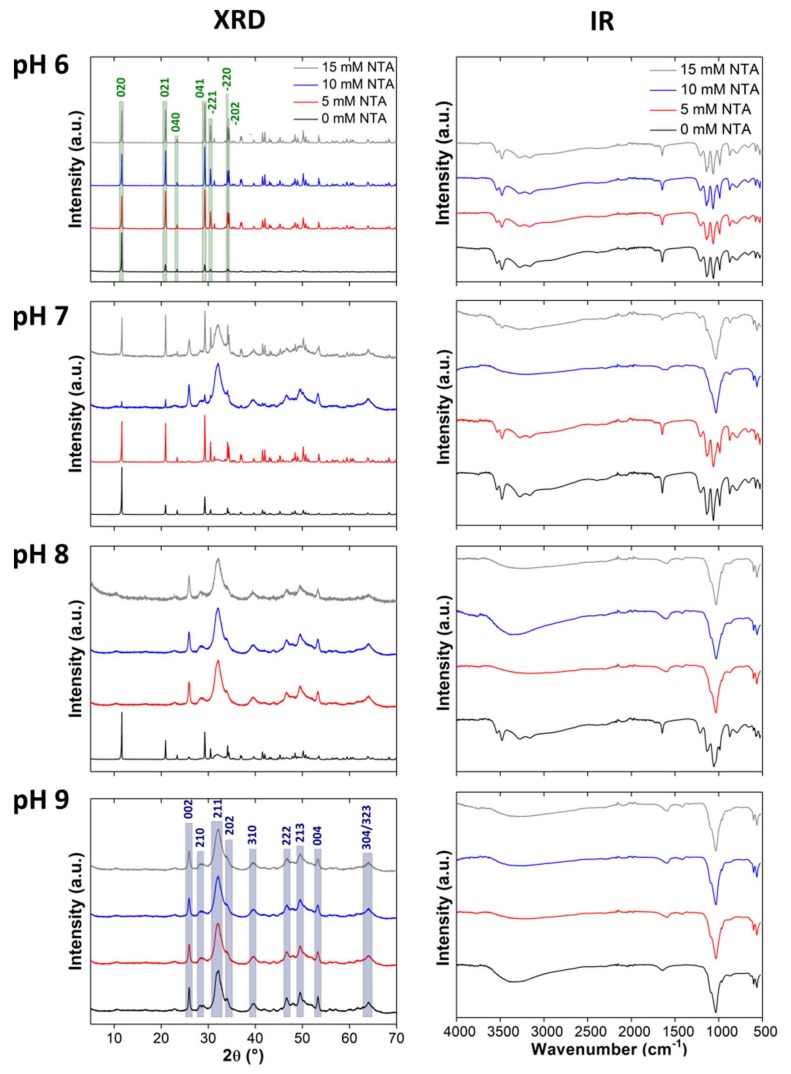
X-ray diffraction (XRD) patterns and infrared (IR) spectra of samples mineralized at different NTA concentrations (0, 5, 10, 15 mM) and pH (6–9). The different intensities in the patterns of the control samples are likely due to sample orientation effects. Highlights indicate dicalcium phosphate dihydrate (DCPD; green) and hydroxyapatite (HAP; blue) signals, respectively. These apply to all images in the manuscript; only the most important reflections are highlighted. a.u.: Arbitrary units.

Figure 2 shows the corresponding XRD and IR spectroscopy data of the precipitates. All samples mineralized at pH 6 produce identical diffractograms. The most intense reflections are at 11.6 (020), 20.9 (021), 29.3 (041), 30.5 (−221), 34.1 (−220), and 34.4 (−202) ° 2θ and less intense reflections can further be observed at 23.4 (040) ° 2θ and at larger 2θ values. All reflections can be assigned to DCPD (ICDD 00-009-0077, reflection assignments given in brackets above).

For samples grown at pH 7, the diffractograms of the control samples and samples grown at 5 mM of NTA show the reflections described for the samples obtained at pH 6; these samples thus are again DCPD. At higher NTA concentrations of 10 and 15 mM, the same DCPD reflections at 11.6 (020), 20.9 (021), 23.4 (040), 29.3 (041), 30.5 (−221), and 34.1 (−220) ° 2θ are observed again. However, these diffractograms also show HAP (ICDD 00-001-1008) reflections at 25.9 (002), 28.5 (210), 31.9 (211), 33.9 (202), 39.6 (310), 46.6 (222), 49.5 (213), 53.2 (004), and 64.1 (304/323) ° 2θ. Furthermore, the relative intensity of the reflections suggests that at an NTA concentration of 10 mM, more HAP than DCPD and at 15 mM more DCPD than HAP is present.

Control samples grown at pH 8 again show the reflections described above indicating the presence of both DCPD and HAP in these samples. All other powders (pH 8 in the presence of NTA and all samples obtained at pH 9) only show reflections indicative of HAP.

In accordance with the XRD data, all samples grown at pH 6 show identical IR spectra. The bands at 3535, 3483, 3273, and 3153 cm^−1^ can be assigned to the O–H stretching vibration of crystal water in DCPD [60,61,62,63]. These bands appear as two intense doublets; this is typical for DCPD and indicates that the water molecules in the DCPD unit cell are bound differently. The doublet at higher wavenumbers (3535 and 3483 cm^−1^) is more intense than the doublet at lower wavenumbers (3273 and 3153 cm^−1^). The more intense doublet is caused by loosely bound water molecules in DCPD and the less intense doublet is caused by water molecules directly bound to calcium ions [64,65,66,67].

The O–H bending vibration of water is observed at 1649 cm^−1^ [60,61,63]. The band at 1209 cm^−1^ stems from the O–H bending vibration in HPO_4_^2−^ [60,61,63,67,68]. The band at 1136 cm^−1^ stems from degenerate ν‘_6_ and ν‘‘_6_ stretching vibrations of HPO_4_^2−^ in DCPD [61,62]. The ν_3_ stretching vibration band of the PO_4_^3−^ group is located at 1065 cm^−1^ and the symmetrical ν_1_ stretching vibration band of P–O(H) in the PO_4_^3−^ group is at 987 cm^−1^ [61,62]. The symmetrical stretching vibration of HPO_4_^2−^ in DCPD can be found at 874 cm^−1^ [60,61]. Bands at 793 and 661 cm^−1^ are caused by the libration vibration of water. The P–O ν_4_ bending vibration is located at 579 and 528 cm^−1^ [60,61]. The same assignment applies to the spectra of the control samples obtained at pH 7 and 8 and the spectra of the samples grown at pH 8 with 5 mM of NTA.

The samples precipitated at pH 7 and 10 mM NTA, at pH 8 and 5, 10, and 15 mM NTA, and at pH 9 and 0, 5, 10, and 15 mM NTA show different IR bands. Bands at 1455 and 1421 cm^−1^ are caused by the ν_3_ stretching vibration and the band at 874 cm^−1^ by the ν_2_ bending vibration of CO_3_^2−^ [60,69,70,71]. This indicates the presence of carbonate ions in the crystal. A band at 1032 cm^−1^ and a shoulder at 1090 cm^−1^ can be assigned to the triply degenerate antisymmetrical O–P–O ν_3_ stretching vibration of PO_4_^3−^ [25,69,71,72]. The symmetrical O–P–O ν_1_ stretching vibration can be found as a shoulder at 962 cm^−1^ [25,72]. Bands at 602 and 563 cm^−1^ are caused by the triply degenerate O–P–O ν_4_ bending vibration of PO_4_^3−^ [25,71,72]. Bands at 1032, 602, and 563 cm^−1^ are characteristic for HAP [71,73].

Finally, the spectra of the control samples mineralized at pH 9 show an additional band at 1641 cm^−1^ and a broad band between 3000 and 3500 cm^−1^. These bands are attributed to adsorbed water [74,75]. The precipitates grown with 15 mM of NTA at pH 7 also show bands that are typical for DCPD and HAP in analogy to the above assignments.

**Figure 3:**
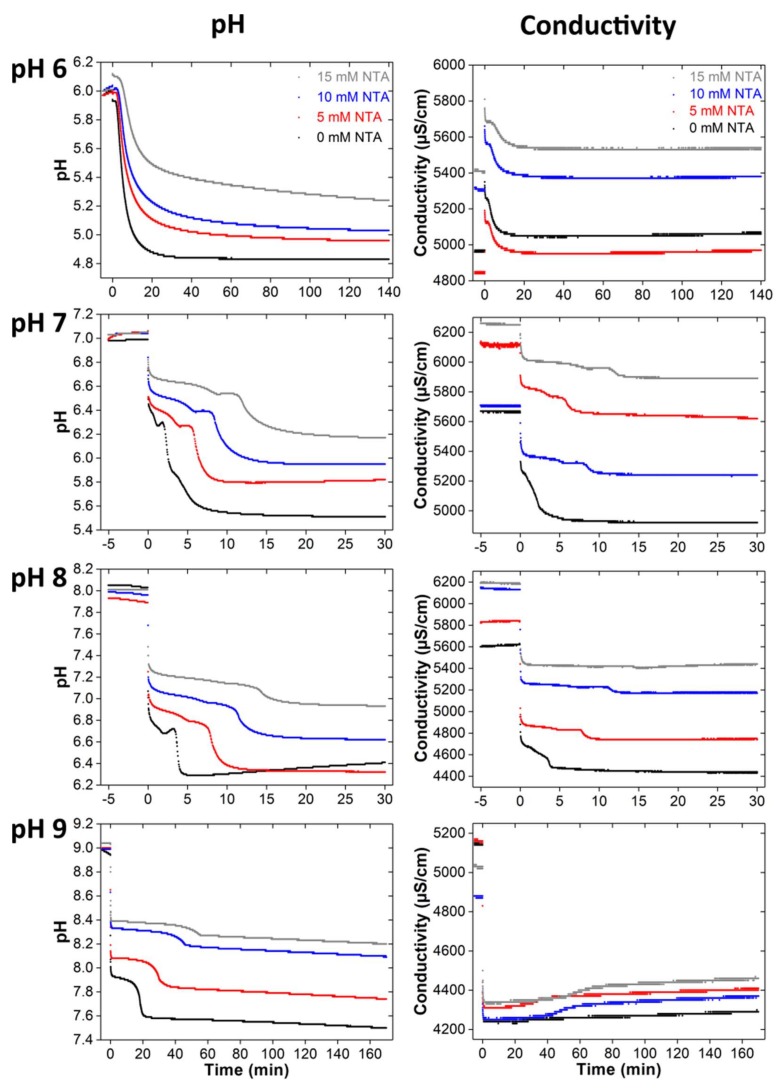
pH and conductivity data obtained from in situ measurements during mineralization with different NTA concentrations and starting pH. Note that the mineralization times are different: the end points (points after which no more changes in the data are observed) of the individual series of measurements were determined from longer measurements (data not shown). At the beginning of each measurement, the pH of the initial solution (40 mM calcium nitrate solution with 0, 5, 10, and 15 mM of NTA) was stirred and the pH and conductivity were recorded for 5 min. Then a 40 mM diammonium hydrogen phosphate solution was added and the mineralization experiment was started. This point is the “0” on the time axis.

To better understand the mineralization process, in situ pH and conductivity measurements were performed (Figure 3). The conductivity and pH data show the same trends but the conductivity data are less clearly resolved. Therefore, only the pH data will be discussed in detail. The general arguments made for the pH curves, however, also apply to the conductometry data.

After phosphate addition, the pH-curves show, regardless of the particular experimental conditions, a general behavior which is characterized by an overall increase in the proton concentration, i.e., an overall decrease of the pH. However, during the mineralization reaction, the pH does not drop linearly but follows a specific pattern. The similarity of the shape of the pH and conductivity curves is reasonable because the protons are strong contributors to the conductivity as they possess the highest specific conductivity of all ions involved in the reaction.

The pH curves recorded for the mineralization at pH 6 in principle show a featureless and almost exponential decrease. At the beginning of the reaction, the pH is around 6.00 and only slightly increases during the first 5 min (−5–0 min, with “0” marking the addition of the phosphate and thus the start of the precipitation reaction). After the addition of the phosphate component, the pH slightly falls for all NTA concentrations except for 15 mM NTA where the pH slightly increases. The start of the CP precipitation is marked by an abrupt pH drop, which shifts to longer reaction times and becomes less pronounced with increasing NTA concentration. After this first significant pH drop, the following pH decrease is less pronounced; it also lasts longer with increasing NTA concentration. From this point on until the end of the experiment after 140 min, the data only show a very weak further pH decrease. Overall, the final pH is higher with higher NTA concentration.

The pH curves observed during the mineralization at a starting pH of 7 exhibit a different shape. The addition of phosphate leads to a pH drop and a minimum. This drop is significantly slower and occurs over a longer time with increasing NTA concentration until the minimum is observed. Then the pH increases again, reaching a maximum. With increasing NTA concentration, the individual features such as the minimum are less pronounced and the maximum gradually becomes a plateau. This plateau exists longer at higher NTA concentrations and is followed by a pH drop and a second, weaker drop. The first pH drop is more pronounced at lower concentrations; the second, weaker pH decrease occurs over longer times at higher NTA concentrations.

The pH curve obtained from the control sample at pH 8 shows (like the control samples formed at pH 7) a minimum and a maximum. Addition of NTA changes the pH curve: similar to the mineralization in the presence of NTA at pH 7, a plateau forms. It is less pronounced at pH 8 than at pH 7 and gradually disappears at higher NTA concentrations. At 15 mM of NTA, no plateau is visible at all. As described above, the plateau is first followed by a pH drop and then a slower pH decrease.

The pH curves obtained from the mineralization reaction at pH 9 are similar to the curves recorded at pH 8 and show a first significant pH drop after phosphate addition. Again, this drop is less pronounced at higher NTA concentrations. Then a plateau (that is more pronounced with higher NTA concentration) is followed by a pH drop and finally the pH-value decreases further.

### 3.2. Clacium Phosphate Mineralization with EDTA

**Scheme 2:**
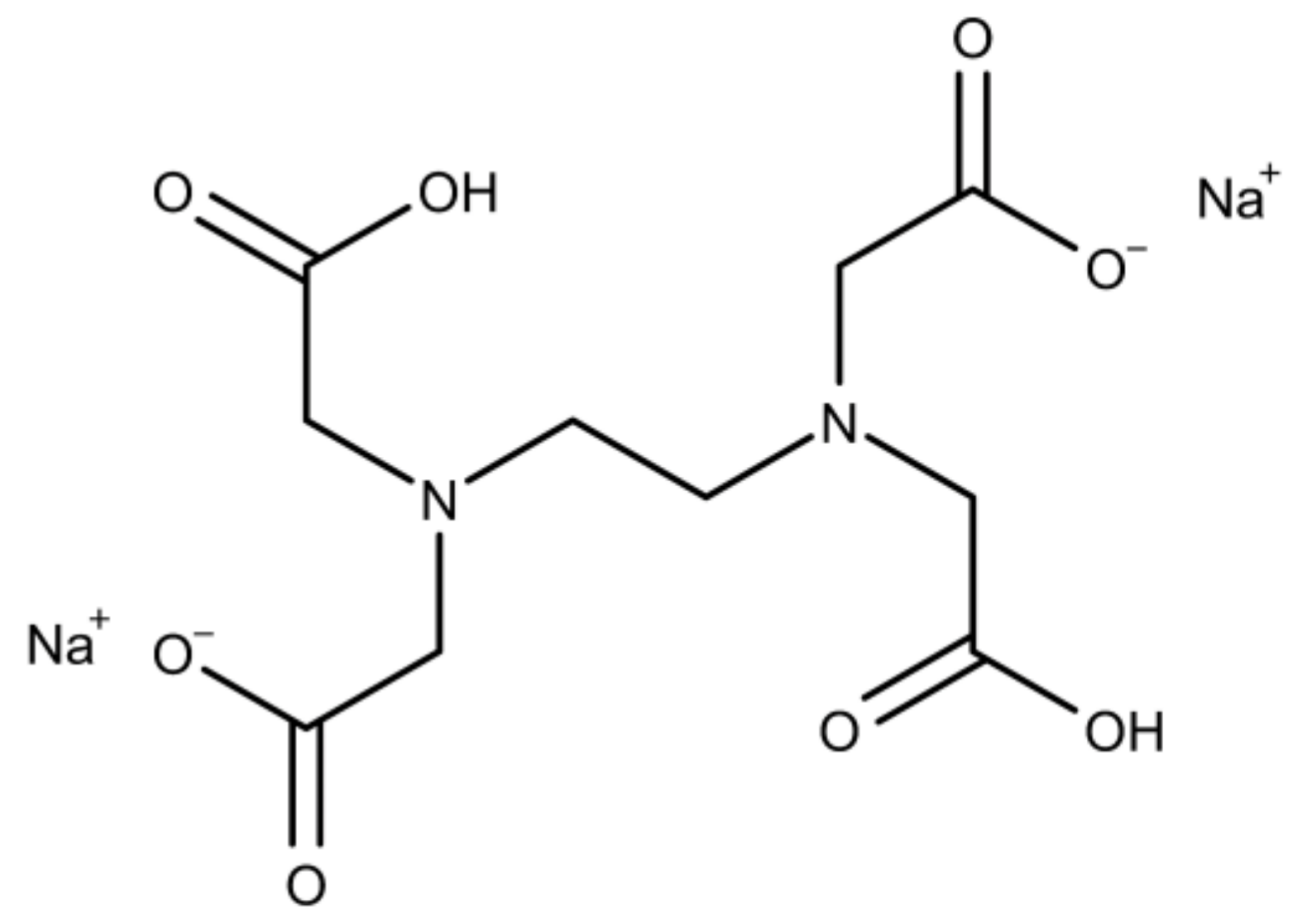
Chemical structure of EDTA [59].

EDTA (Scheme 2) is a hexadentate ligand able to form chelate complexes with Ca^2+^. Calcium phosphate mineralization with EDTA was done as described for NTA (pH 6–9, EDTA concentrations of 0, 5, 10, and 15 mM, and 4 h reaction time).

**Figure 4:**
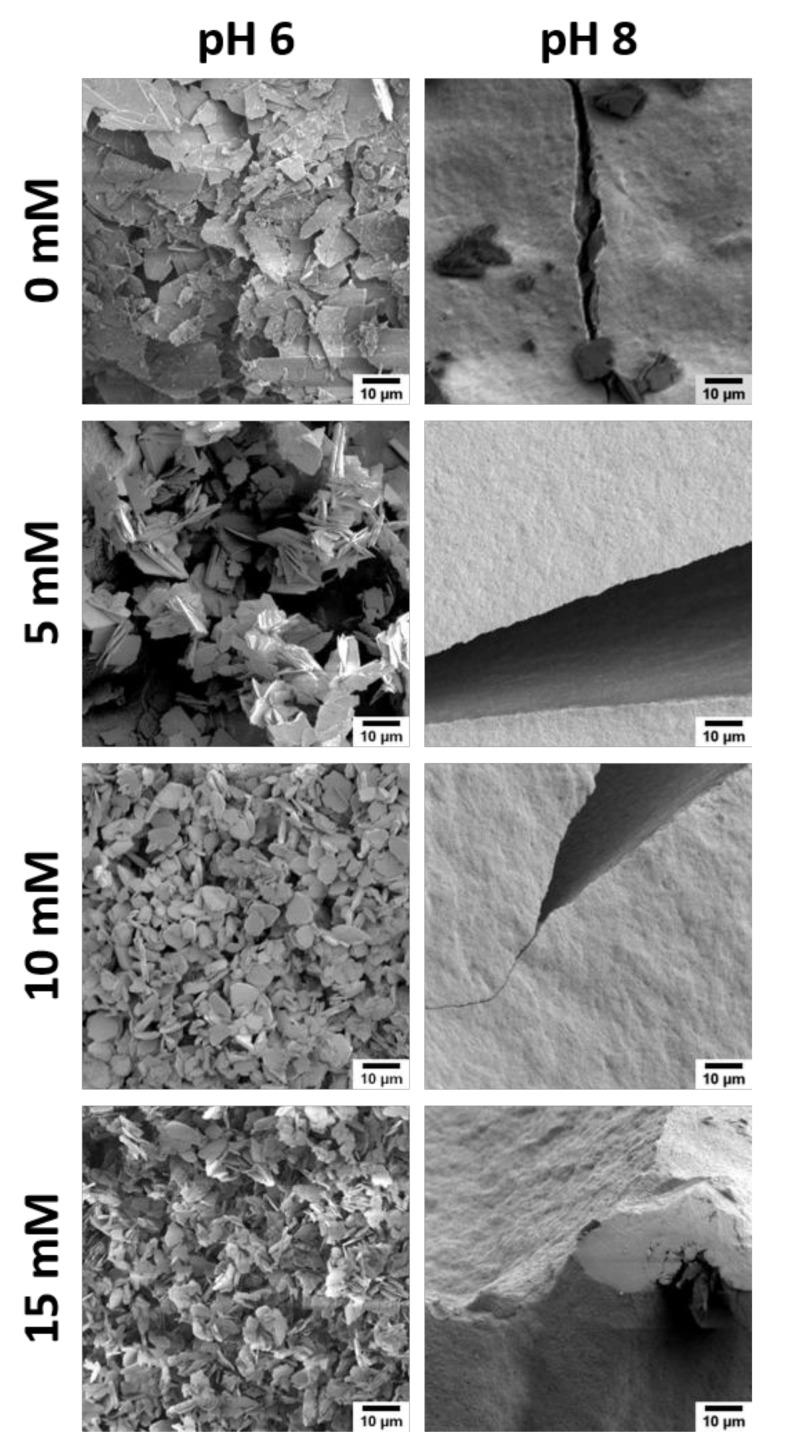
Representative SEM images of CP powders grown with EDTA. Note that the images of the control samples (grown without additive) are identical to those shown in Figure 1.

Figure 4 shows SEM images of some precipitates. The particle morphologies are quite similar to those shown above (Figure 1) and hence, only the images of samples made at pH 6 and 8 are shown. Again, the control samples obtained at pH 6 consist of plates of different sizes; the small particles are on the order of 5–7 µm and the bigger plates around 13–16 µm.

Samples mineralized at pH 6 and 5 mM of EDTA have sizes that are similar to the control sample (5–16 µm). The particle morphologies are quite similar as well, although SEM suggests that the particles grown in the presence of 5 mM of EDTA have a somewhat better-defined crystal habit with clearer edges and well-developed faces. Particles grown with 10 mM of EDTA contain smaller plates (2–9 µm) and are more rounded. The plates obtained at 15 mM of EDTA are very similar to the samples obtained at 10 mM of EDTA.

Similar to the samples grown with NTA (Figure 1), powders grown at pH 8 are similar, irrespective of the EDTA content. While the control sample contains plates and micrometer-sized blocks, the precipitates grown in the presence of EDTA only show large blocks. Identical to samples grown with NTA, these blocks consist of densely packed nanoparticles and break upon drying.

**Figure 5:**
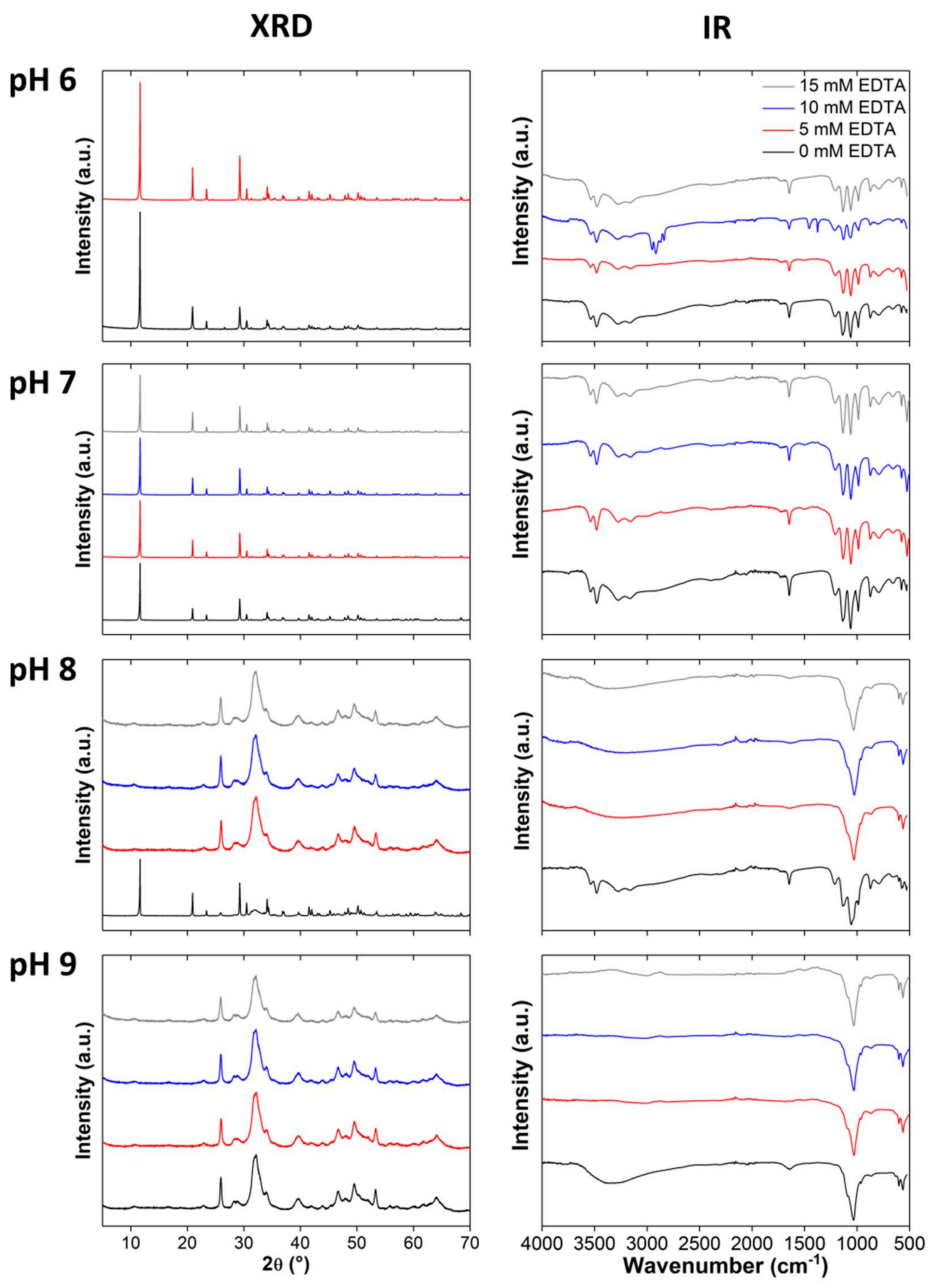
XRD patterns and IR spectra of samples precipitated at different EDTA concentrations (0, 5, 10, 15 mM) and pH (6–9). a.u.: Arbitrary units.

Figure 5 shows XRD patterns of the precipitates. At pH 6 and high EDTA content (10 and 15 mM), only very small amounts of precipitate were obtained; hence no XRD analysis of these materials was possible. The XRD patterns of samples grown at pH 6 (0 and 5 mM EDTA) and 7 (0, 5, 10, and 15 mM EDTA) show DCPD (ICDD 00-009-0077) reflections at 2θ of 11.6 (020), 20.9 (021), 23.4 (040), 29.3 (041), 30.5 (−221) und 34.1 (−220) ° 2θ.

Calcium phosphate mineralized at pH 8 with EDTA (5, 10, and 15 mM) show reflections at 25.9 (002), 28.5 (210), 32.1 (211), 33.9 (202), 39.6 (310), 46.6 (222), 49.5 (213), 53.2 (004) und 64.1 (304/323) ° 2θ which are typical for HAP (ICDD 00-001-1008). As stated above, the control samples obtained at pH 8 are a mixture of DCPD and HAP. At pH 9, only HAP forms, similar to the samples grown with NTA.

Infrared spectroscopy (Figure 5) supports the XRD assignments. Overall, the XRD and IR assignments are identical to those given for the samples grown with NTA above. In short, samples mineralized at pH 6 and 7 with 0, 5, 10, and 15 mM EDTA show characteristic DCPD bands at 3537, 3271, 3159, 1649, 1207, 1136, 1061, 987, 793, 660, 577, and 528 cm^−1^. Products mineralized at pH 7 with 10 mM EDTA also show additional bands at 2951, 2916, 2871, 2839, 1452, and 1377 cm^−1^ from EDTA. Bands between 2800 and 3000 cm^−1^ can be assigned to chelating OH or OH stretching vibrations in the presence of hydrogen bonds. The band at 1452 cm^−1^ is caused by the R–CH_2_–R scissor vibration. The symmetric stretching vibration at 1377 cm^−1^ stems from CO_2_^−^ of the EDTA salt [76].

The control samples grown at pH 8 exhibit typical bands for DCPD and HAP. The spectra of the samples mineralized at pH 8 in the presence of EDTA and all spectra of the samples mineralized at pH 9 can be attributed to HAP. The only additional features are bands in the spectra of powders mineralized at pH 8 and the control sample mineralized at pH 9. Here, bands between 1634 and 1641 cm^−1^ are likely caused by adsorbed water. Furthermore, these spectra also show a band between 3000 and 3500 cm^−1^, attributed to the OH stretching vibration of water or EDTA.

**Figure 6:**
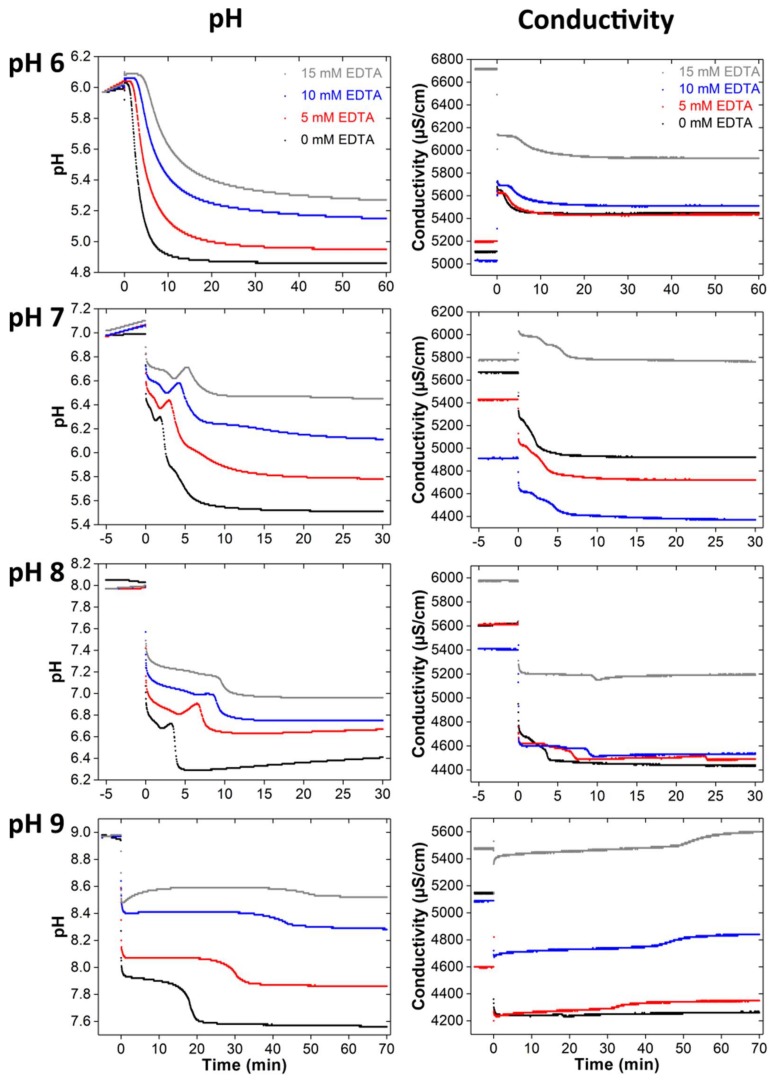
pH and conductivity curves of samples mineralized at different EDTA concentrations (0, 5, 10 and 15 mM) and starting pH (6–9). Note that the mineralization times are different. Long time measurements have shown that the values do not change anymore after the reaction times shown here; the corresponding data are therefore not shown.

Figure 6 shows the corresponding pH and conductometry data for different starting pH and EDTA concentrations. As stated above for the powders grown with NTA, the pH curves again have a much better resolution than the conductivity data and we will thus focus on these data.

The pH curves of the samples grown at pH 6 are similar. After phosphate addition, the pH slightly decreases in the solutions containing 0 and 5 mM of EDTA; conversely, it slightly increases in the solutions with 10 and 15 mM of ETDA. Subsequently a plateau is observed; it persists longer and is more pronounced at higher EDTA concentrations. After the plateau, the pH strongly decreases. This drop is less clear with increasing EDTA concentration. Thereafter, the pH decreases more slowly until the end of the reaction.

The pH curves recorded in the reactions starting at pH 7 show the same features. After phosphate addition, the pH rapidly decreases and then less steeply until a minimum is reached. In addition, an EDTA concentration dependence is visible: the minimum shifts to longer mineralization times and higher pH values with increasing EDTA concentration. After the minimum, the pH increases to a maximum, which again shifts with increasing EDTA concentration. Then, a sharp pH decrease follows; this decrease is more pronounced with lower EDTA concentrations. Additionally, a shoulder is visible for EDTA concentrations of 0 and 5 mM. Finally, the pH slightly drops and then tails off.

The pH curves obtained at pH 8 are somewhat different: they all show a first rapid drop after phosphate addition, then go through a minimum followed by a secondary maximum. Thereafter, the pH drops rather steeply and then reaches a constant value towards the end of the reaction. Again, there is a clear influence of the EDTA concentration leading to a general shift of the pH curves to higher pH with higher EDTA concentrations. Moreover, the individual features just described (minimum, maximum) are less clearly visible at higher EDTA concentrations.

The pH curves obtained from the reactions at pH 9 are different. The pH quite drastically drops immediately after the start of the mineralization; this drop is more pronounced at lower EDTA concentrations. After passing through a slight minimum at higher EDTA concentration, a rather broad plateau is visible. This plateau finally levels off after a second drop.

## 4. Discussion

Although NTA and EDTA are chemically and structurally similar, the effect of the additives on the particle morphologies, sizes, and crystal phases of the precipitates are somewhat different. X-ray diffraction and IR spectroscopy (Figure 2 and Figure 5) show that mainly DCPD and HAP precipitate. Moreover, there is a clear correlation between crystal phase, pH, and additive concentration: low pH and low additive concentrations lead to DCPD while higher pH and higher additive concentrations lead to HAP. This effect is more pronounced for NTA in the sense that NTA is the additive that appears to drive the transformation to HAP more effectively.

As a result, the current data show that the solution pH is not the only decisive factor controlling the formation of a certain crystal phase. Rather, the additive appears to affect the local environment for mineral precipitation and phase transformation, similar to data by Bigi and coworkers [13,14,16,22,29]. We currently speculate that the addition of the additives influence the local pre-nucleation aggregation and/or the structure of the early CP clusters; this may in turn affect the formation of the final crystal shape and phase, similar to existing examples [77,78].

Besides the crystal phase, the additives also affect the crystal morphology (Figure 1 and Figure 4). The control samples obtained at pH 6 and 7 and partially at pH 8 show typical DCPD plates. Samples mineralized with NTA contain rods; these materials are still DCPD, but rods are quite an unusual morphology for DCPD. EDTA has a weaker influence on the morphology. Rather than rods, EDTA favors the formation of rounded DCPD plates with a reasonably narrow diameter distribution. Overall, both additives are therefore simple tools to tune the morphology from conventional DCPD plates [36,48,49,60,63,68,79,80] to potentially more useful shapes; especially the rods lend themselves to application in biomaterials as they may provide access to DCPD-based composites with interesting properties, e.g., mechanical properties.

Higher additive concentrations and higher pH lead to large, rather compact blocks consisting of small, densely packed nanoparticles, which is consistent with the literature [80]. It also matches the XRD data just discussed: the plate, rounded plate, and rod morphologies observed by SEM correlate with the appearance of DCPD signals in the XRD and IR data. In contrast, the highly aggregated nanoparticle blocks correlate with the respective HAP signals. Samples exhibiting both plate-like features and aggregated blocks of nanoparticles also show DCPD and HAP signals in XRD and IR spectra; these samples thus contain DCPD plates and HAP nanoparticles.

Additive-induced crystal structure and morphology changes of CP are known [12,16,20,33,35,81,82] but there is no study on the effects of NTA and EDTA. The data suggest that both additives adsorb on the crystal surface and change the final particle morphologies. As EDTA leads to rounded particles, we presume that EDTA prevents the growth of the DCPD plates along the *a*- and the *c*-axis in a roughly equal manner; this reduces the growth in both directions and leads to the rounded plates observed here. In contrast, NTA reduces the growth either along the *a*- or the *c*-axis much more strongly leading to the observed rod-like morphology, where the growth along the least blocked face is favored. Indeed, such processes are known [29,83,84,85]. Strong effects of additives on the formation on the transition of spherical to rod-like particles have recently been calculated as well [86].

In contrast to the effect of the additive of both NTA and EDTA on the DCPD particles, the effect on the HAP particles is rather uniform: in all cases thick, dense blocks consisting of nanometer-sized particles could be observed, similar to previous studies [82,87]. It must, however, clearly be noted here that the phase transition from DCPD to HAP is not only driven by the solution pH but also by the additive. Overall, there is a clear trend: low pH and low additive concentrations favor the formation of DCPD (where the morphology changes with increasing additive concentration) and the higher the pH and the additive concentrations are the more effectively HAP forms. This suggests that effects besides additive adsorption on the growing crystals come into play. 

For example, it is possible that the transformation of DCPD to HAP at higher additive concentration is favored at higher pH because at higher pH the additives are less protonated. As the transition from DCPD to HAP releases protons [24,49,88], the higher number of charged carboxylate groups may favor the transformation by taking up some of the released protons, similar to some other examples [37,38,39,89,90]. In contrast, at lower pH, the additives show a higher degree of protonation and thus the DCPD/HAP transformation is slower [35]. Such a model is consistent with an earlier study [88] but difficult to prove unambiguously. Additionally, the effects of (intermediate) complex formation must be considered. Complexation will reduce the amount of free calcium and thus, according to the Ostwald step rule, delay the nucleation of thermodynamically more stable phases.

The above data therefore support the previous assumption that the mineralization is driven by three main effects: (i) the solution pH; (ii) a protonation/deprotonation equilibrium [88]; and (iii) the complexation of calcium by NTA and EDTA. Moreover, the adsorption of additives to existing crystalline or amorphous CP particles also stabilizes intermediate (crystal) phases [42]; this is clearly visible in the pH vs. time data (Figure 3 and Figure 6): the higher the additive concentration, the slower the formation of the final product. Similar to a study by Chen et al. [42], our data suggest that initially amorphous calcium phosphate (ACP) precipitates directly after mixing of the calcium and phosphate solutions; this process is accompanied by a strong pH drop. This step is followed by the formation of DCPD (the plateau) and finally the last pH drop indicates the start of the HAP crystallization. Such a process was also observed by other groups [91,92].

Moreover, Chen et al. [42] showed that the induction time for HAP nucleation increases in the presence of citrate (and strong effects of citrate have also been reported for calcium carbonate mineralization [93]); IR spectroscopy data suggest that this is due to a close association of the citrate with the ACP. Hu et al. [45] reported that the carboxyl groups of the citrate are deprotonated at pH 7.5–8.0 and can therefore also strongly adsorb on the HAP (not the ACP) surface. The adsorption of the citrate therefore prevents HAP crystal growth and the adsorbed citrate molecules yield a negative surface charge. According to the authors, this results in the repulsion of further phosphate ions, which in turn stops the crystal growth and also prevents the aggregation of the HAP crystals [45]. Likely, a similar explanation applies to the current study. For example, all pH curves show a longer induction time at higher additive concentration. This indicates that a higher additive concentration provides a better stabilization of the intermediates and slows down the phase transitions.

Interestingly, NTA appears in many cases to be a more effective stabilizer for the intermediate phases than EDTA. This is somewhat counterintuitive because EDTA provides more Ca^2+^ binding sites. However, considering the proton sponge argument introduced above [88], it might be possible that EDTA is able to trap protons more effectively which again can be released during the further mineralization reaction. In accordance with the earlier publication, this would then also favor the phase transition rather than inhibit it.

## 5. Conclusions

Both NTA and EDTA are efficient growth modifiers for CP mineralization, but they act differently. With NTA rod-like DCPD crystals and with EDTA rounded DCPD plates form at low pH. In contrast, the morphology of the HAP powders obtained at higher pH is not affected by the two additives. The formation of the final powders is a multistep process with different precursor and intermediate phases. The formation of the precursor phases and the individual phase transitions to the final products depend on the pH, the additive, and the additive concentrations. The phase transitions are delayed in the presence of NTA and EDTA in a different manner but in both cases the delay is longer with increasing additive concentration. Overall, the data show that even simple additives can be highly efficient control agents for tuning the outcome of CP mineralization.

## Figures and Tables

**Table 1:** Concentrations, masses, and volumes used for calcium phosphate (CP) precipitation.

Reaction Vessel	Ca(NO_3_)_2_		NTA or EDTA		(NH_4_)_2_HPO_4_		Solution after Mixing	
	c (mM)	V (mL)	c (mM)	m (g)	c (mM)	V (mL)	c(Ca), c(P_i_) (mM)	V (mL)
1	40	50	0	0 ^a,b^	-	-	20	100
A	-	-	-	-	40	50		
2	40	50	5	0.138 ^a^ 0.186 ^b^	-	-	20	100
B	-	-	-	-	40	50		
3	40	50	10	0.275 ^a^ 0.372 ^b^	-	-	20	100
C	-	-	-	-	40	50		
4	40	50	15	0.413 ^a^ 0.558 ^b^	-	-	20	100
D	-	-	-	-	40	50		

Numbered solutions (1, 2, 3, 4) and solutions labeled with a letter (A, B, C, D) denote calcium-containing solutions and phosphate solutions, respectively. ^a^ Denotes nitrilotriacetic acid (NTA) and ^b^ denotes ethylenediaminotetraacetic acid (EDTA) masses in the solutions.

**Table 2:** Concentrations, masses, and volumes used for pH and conductivity measurements during CP precipitation.

Reaction Vessel	Ca(NO_3_)_2_		NTA or EDTA		(NH_4_)_2_HPO_4_		Solution after Mixing	
	c (mM)	V (mL)	c (mM)	M (g)	c (mM)	V (mL)	c(Ca, P_i_) (mM)	V (mL)
1	40	15	0	0 ^a,b^	-	-	20	30
A	-	-	-	-	40	15		
2	40	15	5	0.041 ^a^ 0.056 ^b^	-	-	20	30
B	-	-	-	-	40	15		
3	40	15	10	0.080 ^a^ 0.112 ^b^	-	-	20	30
C	-	-	-	-	40	15		
4	40	15	15	0.124 ^a^ 0.168 ^b^	-	-	20	30
D	-	-	-	-	40	15		

Numbered solutions (1, 2, 3, 4) and solutions labeled with a letter (A, B, C, D) denote calcium-containing solutions and phosphate solutions, respectively. ^a^ Denotes NTA and ^b^ denotes EDTA masses in the solutions.

**Figure A1:**
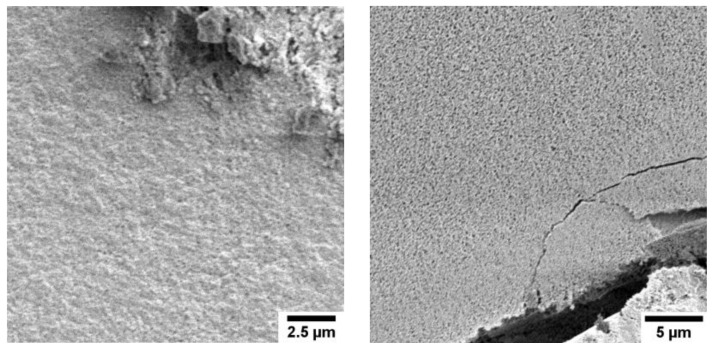
High magnification SEM images of samples mineralized at pH 8 using 5 mM NTA (**left**) and 10 mM NTA (**right**) as additive.
